# Identification of performance drivers for an antibody producing CHO-S cell line culture in the Allegro™ XRS 20 single-use bioreactor utilizing historical data

**DOI:** 10.1186/1753-6561-9-S9-P33

**Published:** 2015-12-14

**Authors:** Todd Sanderson, Alex Martino, Ing-Kae Wang, Amanda Rose, Byron Rees, Lisa Bradbury

**Affiliations:** 1 Pall Life Sciences, 20 Walkup Drive, Westborough, MA, 01581, USA

## Background

During the development of processes for high production CHO fed batch cultures, there is usually insufficient time for thorough process optimization. Once defined, these processes often have ranges rather than specific values for many parameters, resulting in minor variations in the runs. These small process variations, combined with resultant differences in performance, can be used to further optimize the process. Over the course of one year, eleven 20 L fed batch cultures of an antibody producing CHO-S cell line were generated in the Allegro XRS 20 bioreactor. In addition to normal minor process variation, small process changes occurred due to method optimization efforts or culture timing requirements. An analysis of the combined historical data was undertaken to better understand the specific culture performance drivers, leading to further improvement in culture outputs, specifically final antibody concentration and maximum viable cell density (VCD).

## Materials and methods

Two different Allegro XRS 20 bioreactors were used for the 20 L fed batch runs. A monoclonal antibody producing CHO-S cell line was grown in CD FortiCHO media fed with EfficientFeed C on five consecutive days and glucose as needed. In all cases the final expansion was done in the XRS 20 biocontainer. Table 1 shows a summary of the process conditions applied throughout the experimental runs.

**Table 1:** Allegro XRS 20 fed batch process conditions.

Cell Line:	Industrial suspension CHO producing mAb biosimilar
**Seed:**	0.3 - 0.5 × 10^6 ^viable cells/mL in 5 L CDFortiCHO (Life Technologies)

**Expansion:**	Expand at 2 - 4 viable cells/mL to 14 L

**Feeding strategy:**	CD EfficientFeed C (EFC, Life Technologies), initial feed at 8.0 ± 2.0 M viable cells.mL Additional bolus feeds every 24 hours for a total of 5 feeds (1.2 L/feed)

**Carbon source:**	Glucose kept above >2 f/L (bolus target >5f/L)

**Antifoam C:**	Added as needed

**Aeration rate:**	1.0 L/min

**DO set point:**	O_2 _control at 40%

**pH set point:**	7.2 ± 0.05

**Agitation:**	**5 L: **25 RPM, 5°X, 5°Y; **14 - 20: **30 or 35 RPM, 15°X, 5°Y

**Temperature:**	37°C

## Results

**Figure 1:**
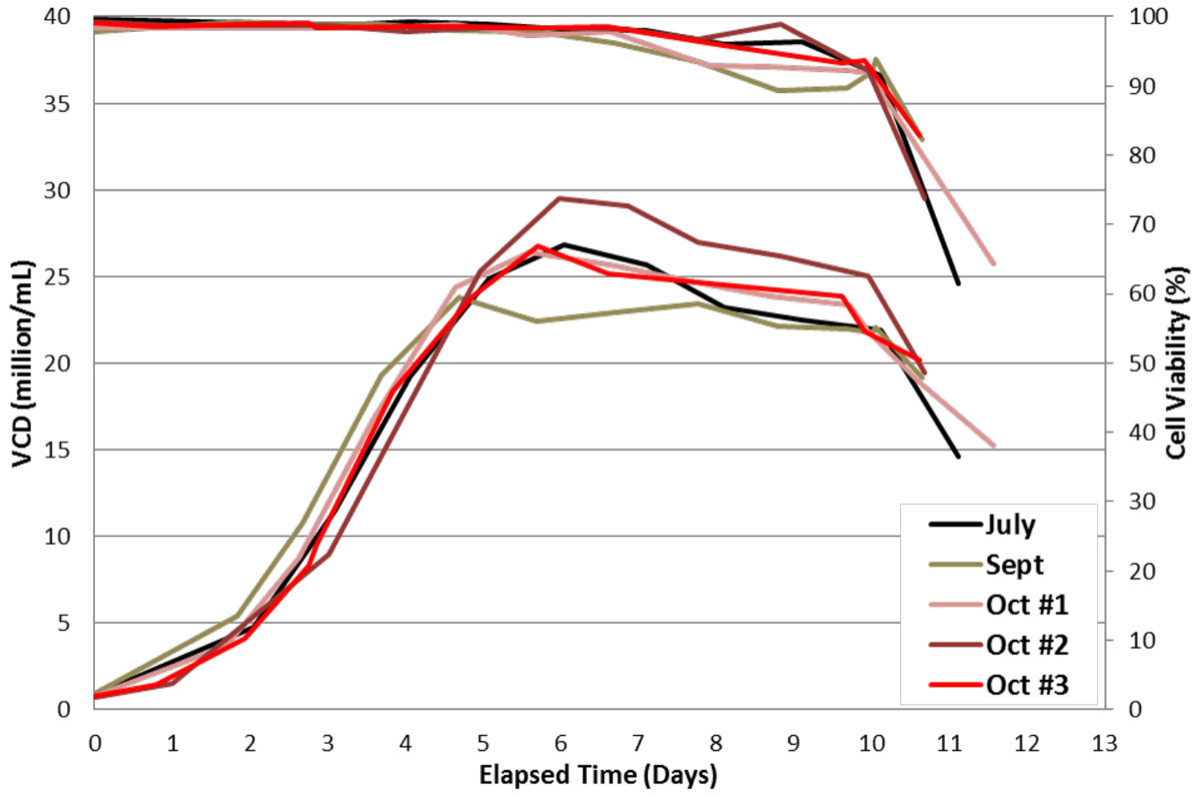
**Viable cell data average of replicate samples (2 or 3)**. Agitation at 30 rpm for all runs.

An initial evaluation of CHO fed batch cell culture in the Allegro XRS 20 bioreactor in terms of growth curve and viability, antibody production, and contaminants demonstrated excellent reproducibility. This provides an opportunity to identify relatively small output differences due to process variation. For the analysis of cell culture performance drivers, the following input variables were considered: passage number; initial and final post expansion seed density; VCD at first feed; agitation; viability at harvest; and total days in culture - data for viable cell density over 5 runs is shown in figure 1. In addition to almost daily sampling for cell count, viability, metabolite/nutrient, gases and pH measures, the final harvest was assayed for mAb, host cell protein (HCP), and DNA concentrations. These outputs were used in the analysis as well. The data from these runs was compiled and analyzed for trends linking the variations in run parameters with the critical outputs of maximum viable cell density and mAb titer.

## Conclusions

The Allegro XRS 20 single use bioreactor generated highly reproducible cell culture performance for this CHO mAb production process, allowing for a holistic analysis of relatively small changes in process conditions and the resultant differences in performance. In three separate paired runs, the first being identical and the second two differing by 30 vs. 35 rpm agitation, the parallel culture results are extremely similar, highlighting the overall reproducibility of the system. This bioreactor reproducibility makes it possible to review historical data to identify performance drivers that were missed in limited optimization testing done at smaller scale prior to the 20 L runs. The VCD at the time of the first feed addition proved very important for overall mAb yield. Although a relationship was not surprising, the optimal range was tighter than previously believed. Nutrient feed timing can be easily modified as there is very good understanding of the growth curves for this CHO-S cell line in the Allegro XRS 20 bioreactor. This opportunistic analysis of historical data has some limitations compared to experiments designed specifically to look at process controls. Despite these limitations the value is clear, it can provide additional process understanding without additional experimentation. Thus, this approach could further understanding of performance drivers for any production run, and can be expanded to other cell line / media combinations in any culture system having sufficient reproducibility to distinguish relatively small changes in process conditions and the resultant small but significant differences in performance.

